# The whole‐brain voxel‐based morphometry study in early stage of T2DM patients

**DOI:** 10.1002/brb3.2497

**Published:** 2022-02-09

**Authors:** Yana He, Liang Li, Jihua Liu

**Affiliations:** ^1^ Department of Medical Imaging First Teaching Hospital of Tianjin University of Traditional Chinese Medicine Tianjin China; ^2^ National Clinical Research Center for Chinese Medicine Acupuncture and Moxibustion Tianjin China

**Keywords:** cognitive impairment, magnetic resonance imaging, type 2 diabetes mellitus, voxel‐based morphometry

## Abstract

**Aim:**

This study aimed to investigate the alterations in whole‐brain gray matter density in early stage type 2 diabetes mellitus (T2DM) patients with cognitive impairment using magnetic resonance imaging.

**Methods:**

Thirty‐six cases of early stage T2DM patients with cognitive impairment (T2DM‐CI), 34 cases of early stage T2DM patients without cognitive impairment (T2DM) and 30 cases of healthy controls (HC) were enrolled. Montreal Cognitive Assessment (MoCA) and Mini‐Mental State Examination (MMSE) scores were used to identify the cognitive impairment. The whole‐brain gray matter density was analyzed using 3D‐T1 BRAVO imaging, using the voxel‐based morphometry method on T1 structure imaging of two groups.

**Results:**

The correlation analysis of total gray matter density with MMSE and MoCA scores in the T2DM‐CI group was performed. There were no significant differences in MMSE and MoCA scores between the HC and T2DM groups. However, the MMSE and MoCA scores in the T2DM‐CI group were significantly reduced compared with the T2DM group. There were no significant differences in age, gender, education, body mass index (BMI) or blood pressure among the three groups. Voxel‐based morphometry (VBM) results showed that the density of left triangle part of inferior frontal gyrus, orbital part of inferior frontal gyrus and opercular part of inferior frontal gyrus and left insula in the T2DM‐CI group decreased compared with the T2DM group. Correlation analysis results showed that there was a significant positive correlation between total gray matter density and scores of MMSE and MoCA scores in the T2DM‐CI group.

**Conclusion:**

In conclusion, total gray matter density is positively correlated with the scores of MMSE and MoCA in T2DM patients, which may be an early sign of cognitive impairment in patients with T2DM.

## Graphical abstract

1. There were no significant differences in MMSE and MoCA scores between the HC and T2DM groups.

2. VBM results showed that the density of left triangle part of inferior frontal gyrus, orbital part of inferior frontal gyrus and opercular part of inferior frontal gyrus and left insula in the T2DM‐CI group decreased compared with those in the T2DM group.

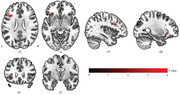

## 1 INTRODUCTION

Diabetes mellitus (DM) is a comprehensive disease involving multiple systems and multiple organ damages, which causes serious harm to human health and affects 463 million people worldwide, according to the 2019 International Diabetes Federation statistics (Khan et al., 2019). Type 2 diabetes mellitus (T2DM) is the most common type of diabetes, accounting for 90%–95% of all cases (W. Xie & Burke, 2020). The incidence of diabetes is increasing especially among the elderly population, reaching 12%–25% in those over 65 years old, with a trend toward younger ages as well. T2DM is a chronic metabolic disorder characterized by insulin resistance and sustained hyperglycemia (Xiao et al., 2021), which are correlated with multiple macrovascular and microvascular complications. These complications, together with chronic inflammation, are important risk factors for accelerated cognitive impairment. The major clinical manifestations are cognitive impairment, dementia, declined short‐term memory, learning ability, information processing speed, attention and executive functions (Spauwen et al., 2013; K. Wang et al., 2008). Approximately 10.8%–17.5% of T2DM patients develop cognitive impairment (Bruce et al., 2003; Hoyer, 1998; Moran et al., 2013). With increasing incidence of diabetes, more attention has been paid to the occurrence and development of associated complications, especially concerning neurocognitive impairment and dementia.

Diabetes can cause changes in the structure and function of the brain. T2DM is directly correlated with cerebral infarction and, in patients with T2DM, counteracts the high signal distribution region of subcortical brain white matter normally observed in individuals without T2DM (Lazarus et al., 2005; Streifler et al., 2003). However, these intracranial changes tend to occur in the later stage of diabetes. Clinical findings show that cognitive impairment can occur at the early stage of diabetes, mainly manifesting as declined memory and learning ability.

The psychological and clinical presentations are the methods used to make a definitive diagnosis of cognitive impairment currently due to the lack of reliable and sensitive biomarkers for distinction (C. Li et al., 2020). Voxel‐based morphometry (VBM) is a classical and fully automated quantitative magnetic resonance imaging (MRI) technique extensively applied to characterize neuropathological changes in the brain (J. Zhang et al., 2015). The relationship between metabolic syndrome, age‐related cognitive decline and gray matter density changes has been studied by VBM (Kotkowski et al., 2019). Studies have also been published to investigate the relationship between T2DM and cognitive impairment through VBM. C. Wang et al. (2014) used VBM to study the neuroanatomical changes in the brain tissue structure of patients with T2DM and analyzed the correlation among those changes and clinical characteristics, cognitive function score and vascular lesions. Y. Zhang et al. (2014) used VBM to study the gray matter density changes in T2DM patients with cognitive impairments. However, all these patients were in the middle and late stages of diabetes, and there is no study on the relationship between gray matter density and clinical presentations. We therefore asked whether there were any alterations of whole‐brain gray matter density in the early stage of T2DM patients with cognitive impairments, and whether gray matter density could be used as a biomarker for T2DM patients with cognitive impairments.

Here, the T2DM patients in the early stage (without complications such as retinopathy, diabetic nephropathy, diabetic foot disease, cerebrovascular disease or recurrent hypoglycemia) with cognitive impairment, as well as controls, were enrolled. Montreal Cognitive Assessment (MoCA) and Mini‐Mental State Examination (MMSE) scores were used to identify the cognitive impairment. The whole‐brain gray matter density was analyzed by VBM. The correlation analysis of total gray matter density with MMSE and MoCA scores was performed. The objective of this study was to reveal the clinical values of VBM for patients at the early stage of T2DM with cognitive impairments, and to explore the potential application of brain gray matter density as a biomarker for cognitive impairment caused by T2DM.

## 2 METHODS

### 2.1 Research participants

A total of 36 cases of early stage T2DM patients with cognitive impairment (T2DM‐CI), 34 cases of early stage T2DM patients without cognitive impairment (T2DM) and 30 cases of healthy controls (HC) were enrolled at the Endocrinology Department of the First Hospital affiliated with Tianjin University of Traditional Chinese Medicine between 2013 and 2018. All participants had received no treatment. This study was approved by the Ethics Committee of First Hospital affiliated with Tianjin University of Traditional Chinese Medicine. The participators recruited for this study were from a community of genetically homogenous individuals of Han Chinese origin. The purpose and method of the current trial were explained to the participants, and informed consent was obtained from all participants. Then, the qualified participants were evaluated and included in the trial.

The inclusion and exclusion criteria for early stage T2DM patients in this study are shown in Table 1. The healthy control (HC) group comprised of healthy volunteers matched by race, sex, age and years of education to the T2DM patients. All participants in the HC group had fasting plasma glucose ≤6.1 mmol/L. There were 30 participants in total, including 16 males and 14 females aged 33–66 years. The exclusion criteria were the same as those for the T2DM patients.

**TABLE 1:** The inclusion and exclusion criteria in this study

The inclusion criteria	The exclusion criteria
Diagnosis of T2DM according to the 2010 Diagnosis and Treatment Guidelines of the American Diabetes Association: 2‐h plasma glucose level ≥11.1 mmol/L during an oral glucose tolerance test (OGTT) and fasting plasma glucose (FPG) ≥ 7.0 mmol/L; taking oral glucose‐lowering drugs with no history of insulin treatment. HbA1c >7%. In the early stages of T2DM (that is, patients without complications such as retinopathy, diabetic nephropathy, diabetic foot disease, cerebrovascular disease, or recurrent hypoglycemia). Years of education ≥5 years. No history of stroke; routine MR examination, such as T1 and T2‐FLAIR sequence, showed no demyelination in brain white matter. No history of hypertension, with systolic blood pressure (SBP) <140 mmHg and diastolic blood pressure (DBP) <90 mmHg. No occurrence of depression, anxiety, schizophrenia, or other psychiatric diseases. Brain atrophy grade of 0—1.	Type 1 diabetes or secondary diabetes. Severe metabolic syndrome, history of stroke and changes in demyelination. History of central nervous system or mental illnesses. Dementia or a family history of dementia. History of traumatic brain injury, tumors, and severe inflammation. Patients with cerebrovascular disease, depression and other factors on brain tissue. History of heavy smoking, alcohol or drug dependence, and use of cognitive improvement, antidepressant or psychiatric drugs. Patients with metallic foreign bodies, claustrophobia and inability to cooperate with MR examination. History of hypoglycemia. Patients with brain atrophy grade >1.

### 2.2 Clinical and laboratory examinations

Clinical and laboratory tests included education years, duration of T2DM years, height, weight, blood pressure, fasting plasma glucose and glycosylated hemoglobin. Body mass index (BMI) was calculated using height and weight.

### 2.3 Cognitive function assessment

In the cognitive function assessment, the MMSE and MoCA were used. The MMSE was composed of 30 items and primarily used to evaluate time and place orientation, computational power, attention, short‐term memory, long‐term memory, language and visuospatial processing. The MoCA test is a widely used screening assessment for detecting cognitive impairment. The identification criteria of cognitive impairment were MMSE score <27 and MoCA score <26.

### 2.4 MRI scan technology

A GE 3.0T MRI scanner (Discovery MR750; General Electric, Milwaukee, WI, USA) with head and neck combined eight‐channel coils was used for data collection. The participants were in the supine position with the head flat and lower jaw closed. The scanning center was positioned 1 cm above the middle of the eyebrows. Special expandable earplugs (3 M‐1100 model, USA) were used for noise shielding.

### 2.5 Conventional MRI scan parameters

The main purpose of T1‐weighted imaging is to determine the extent of brain atrophy. The standard used was a 10‐grade measurement method. The main classification of the 10‐grade method was based on the size of brain ventricles and the width of brain sulci. The normal state was marked as grade 0, while significant atrophy was marked as grade 9. The included participants displayed grades of 0–1, while those with grades >1 were excluded.

T2‐weighted and fluid‐attenuated inversion recovery (T2WI‐FLAIR) sequences were used to exclude intracranial organic and demyelinating lesions. The scan parameters were as follows: time of repeat (TR) = 8000 ms, time of echo (TE) = 145 ms, time of inversion (TI) = 2000 ms, matrix = 256 × 224, field of vision (FOV) = 24 cm × 24 cm, NEX = 1, layer thickness = 5 mm, interval = 1.5 mm, layer numbers = 20 and scan time = 1 min 37 s. All images were evaluated by two experienced radiologists.

### 2.6 3D volume sequence (3D‐T1 BRAVO) imaging

The 3D‐T1 BRAVO scan used the median sagittal plane as the scan baseline, and the sagittal plane was scanned using the 3D‐T1 BRAVO sequence. The scan parameters were as follows: TR = 8.2 ms, TE = 3.2 ms, TI = 450 ms, flip angle (FA) = 12°, matrix = 256 × 256, FOV = 25.6 cm × 25.6 cm, NEX = 1, layer thickness = 1 mm, noninterval scan, layer numbers = 192 and scan time = 5 min 17 s.

### 2.7 Data postprocessing

The computational anatomy toolbox (cat12, 12.5 version) was run using statistical parametric mapping software (SPM12, http://www.fil.ion.ucl.ac.uk/spm). Data postprocessing and analysis of T1 structural images were conducted using default parameters.

### 2.8 Spatial standardization

The 3D‐T1‐weighted brain structure images of all participants were registered to the Montreal Neurological Institute (MNI) standard space, which was previously combined as the initial location, and the standardized voxel size was 1.5 mm^3^.

### 2.9 Segmentation

The standardized brain structural images were effectively segmented into three parts: gray matter, white matter and cerebrospinal fluid.

### 2.10 Image smoothing

The segmented and modulated gray matter images were smoothed with an 8‐mm full‐width at half‐maximum Gaussian kernel. The imaging data after smoothing were more in line with a normal distribution, thus eliminating the error after image reconstruction and facilitating statistical analysis. Using the cat12 toolbox, the density of gray matter, white matter and cerebrospinal fluid of each participant was calculated for the three segmented images.

### 2.11 Statistical analysis of the data

All data are presented as the mean ± standard deviation (*x* ± *s*). SPSS 19.0 was employed for the corresponding analysis. Intergroup comparisons of age, years of education, height, weight, BMI, blood pressure and MMSE and MoCA scores were conducted using Student's *t*‐test followed Mann Whitney test. Sex differences were tested using the *χ* ^2^ test.

The age and sex of all participants were adjusted as covariates, and the gray matter of the whole‐brain was used as a template to conduct the one‐way analysis of variance (ANOVA), followed by a Dunn's multiple comparisons test on the gray matter density of the three groups in the SPM12 software. The initial threshold value was set as *p* < .001, and AlphaSim was used for threshold value correction to extract the brain region clusters >10.

## 3 RESULTS

**FIGURE 1:**
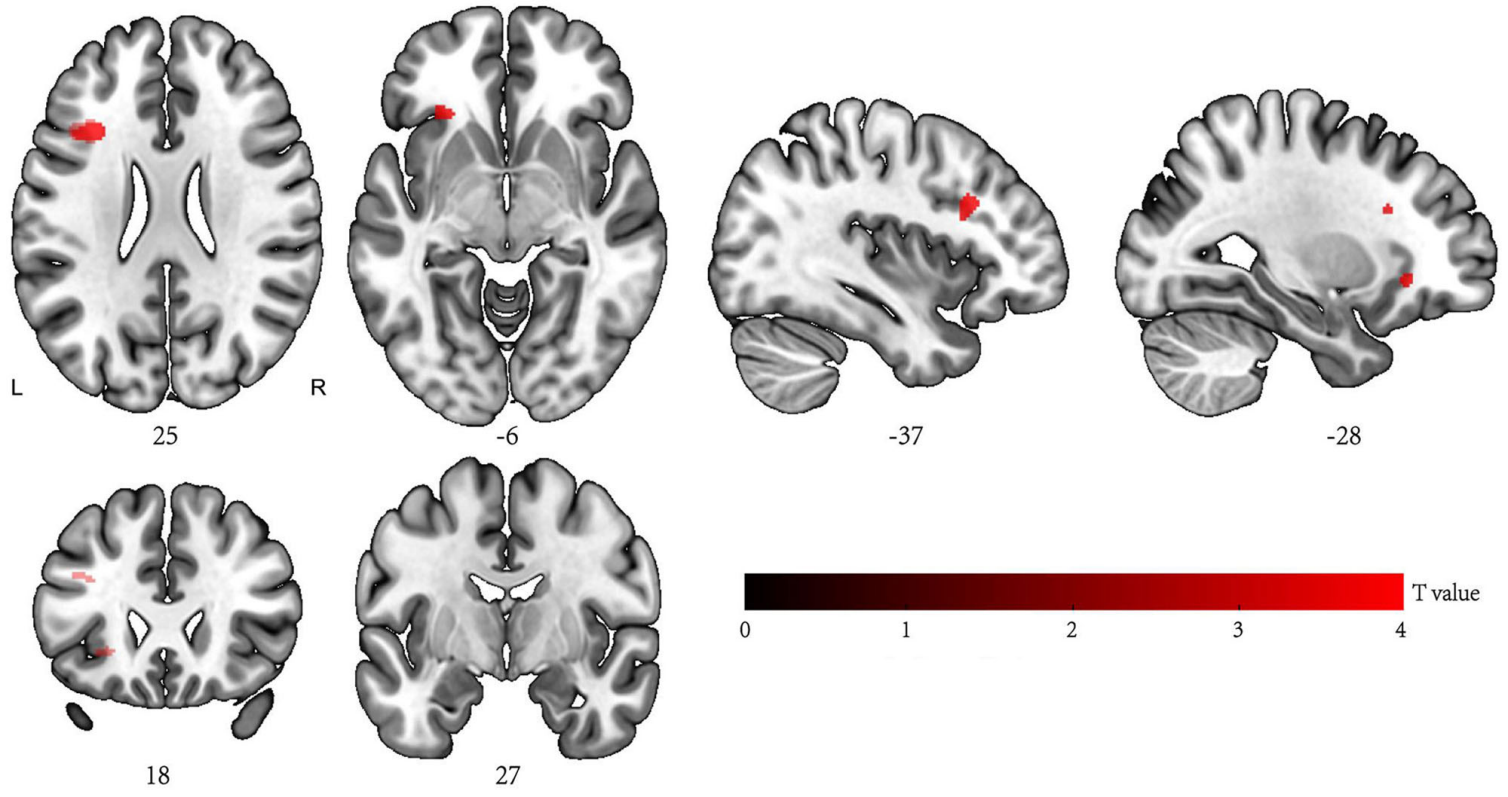
Brain regions in which the gray matter volume decreased in the early stage of type 2 diabetes mellitus with cognitive impairment (T2DM‐CI) compared with early stage of type 2 diabetes mellitus without cognitive impairment (T2DM) by voxel‐based morphometry (VBM). The VBM result showed that the brain regions in the T2DM group were larger than those in the T2DM‐CI group. The volume of the left triangular inferior frontal gyrus and orbital inferior frontal gyrus as well as the operculum inferior frontal gyrus and left insula in the T2DM‐CI group decreased compared with that in the T2DM group, (*p* < .001, cluster >10, the results were not adjusted)

On the basis of strictly controlling the inclusion and exclusion criteria shown in Table 1, head movement control and cognitive function assessment, 36, 34 and 30 cases were included in the T2DM‐CI, T2DM and HC groups, respectively, all of whom were Han Chinese. The T2DM‐CI group included 22 males and 14 females, with ages of 33–67 years, a disease course of 2–6 years and a length of education that ranged from 5 to 18 years. The T2DM group included 19 males and 15 females, with ages of 35–65 years, a disease course of 1–6 years. In the healthy control group, 30 participants (16 males and 14 females) were included, with ages of 33–66 years and the length of education ranged from 7 to 18 years (Figure 1).

**FIGURE 2:**
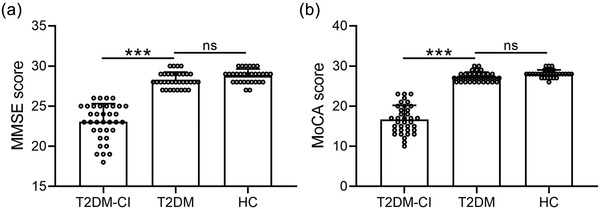
The early stage of type 2 diabetes mellitus with cognitive impairment (T2DM‐CI) showed decreased Mini‐Mental State Examination (MMSE) scores (a) and Montreal Cognitive Assessment (MoCA) scores (b) compared with early stage of type 2 diabetes mellitus without cognitive impairment (T2DM) and healthy control (HC). The data presented are mean ± SD, ***p < .001; ns: no significance. One‐way analysis of variance (ANOVA) followed by a Dunn's multiple comparisons test

There were no significant differences in gender, age, years of education, height, weight, BMI or blood pressure among the T2DM‐CI, T2DM and HC groups. The MMSE and MoCA scores were significantly decreased in the T2DM‐CI group compared with the T2DM and HC groups (Figure 2). However, no significance in either MMSE or MoCA score was observed between the T2DM and HC groups. The demographic indicators, clinical data and cognitive function scores of the three groups are shown in Table 2.

**TABLE 2:** The general data of early stage of type 2 diabetes mellitus with cognitive impairment (T2DM‐CI), early stage of type 2 diabetes mellitus without cognitive impairment (T2DM) and normal healthy control groups

	T2DM‐CI (*n* = 36)	T2DM (*n* = 34)	HC (*n* = 30)	*p*‐Value (T2DM‐CI vs. T2DM)
Age (years)	54.17 ± 9.89	52.39 ± 10.55	53.44 ± 10.21	.758
Gender (male/female)	22/14	19/15	16/14	.657
Education (years)	9.43 ± 4.18	10.26 ± 4.39	11.84 ± 3.98	.194
Duration of T2DM (years)	4.35 ± 1.38	3.93 ± 1.55	–	.671
BMI (kg/m^2^)	24.78 ± 4.12	25.51 ± 3.36	24.43 ± 2.97	.252
SBP (mmHg)	128.66 ± 7.94	126.11 ± 6.16	124.42 ± 6.91	.097
DBP (mmHg)	86.93 ± 5.16	84.79 ± 5.75	83.47 ± 4.94	.394
FPG (mmol/L)	9.55 ± 1.74	8.87 ± 1.39	5.46 ± 0.46	.086
HbA1c (%)	9.48 ± 1.43	8.83 ± 1.26	5.61 ± 0.89	.106
MMSE score	23.06 ± 2.24	28.26 ± 0.96	28.77 ± 0.86	<.001
MoCA score	16.69 ± 3.49	27.29 ± 1.17	28.07 ± 0.94	<.001

*Note*: The data presented are mean ± SD or *n*. The comparisons of data between the T2DM‐CI and T2DM groups were done by unpaired Student's *t*‐test followed Mann Whitney test or *χ* ^2^ test.

Abbreviations: BMI, body mass index; DBP, diastolic blood pressure; FPG, fasting plasma glucose; HC, healthy control; HbA1C, glycosylated hemoglobin; MoCA, Montreal Cognitive Assessment; MMSE, Mini Mental State Exam; SBP, systolic blood pressure.

Compared with that in the T2DM group, the density of the left triangular inferior frontal gyrus and orbital inferior frontal gyrus, as well as the operculum inferior frontal gyrus and left insular lobe, in the T2DM‐CI group was significantly reduced (Table 3, *p* < .001, cluster > 10, the results were not adjusted).

**TABLE 3:** VBM intergroup differences in brain gray matter volume between the early stage of type 2 diabetes mellitus with cognitive impairment (T2DM‐CI) and early stage of type 2 diabetes mellitus without cognitive impairment (T2DM)

	MNI coordinate				
Anatomical location	*X*	*Y*	*Z*	Voxels	Peak *t*‐value
Left triangular inferior frontal gyrus	−36	21	28.5	177	4.638
Left orbital inferior frontal gyrus	−27	28.5	−4.5	25	3.890
Left insular lobe	−27	28.5	−4.5	18	3.890
Left operculum inferior frontal gyrus	−36	21	28.5	10	4.638

Abbreviation: MNI, Montreal Neurological Institute.

**FIGURE 3:**
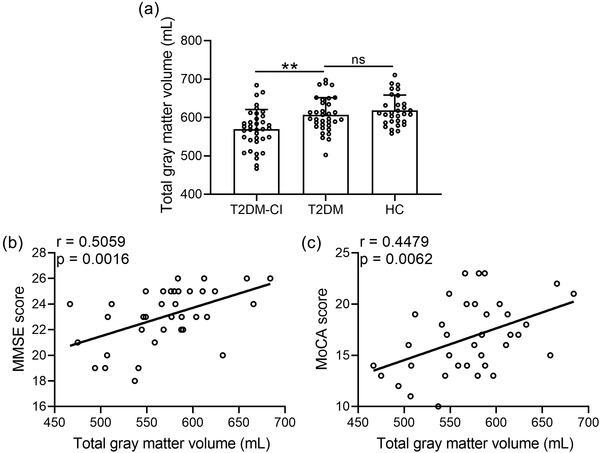
The early stage of type 2 diabetes mellitus with cognitive impairment (T2DM‐CI) showed decreased total gray matter volume (a) compared with early stage of type 2 diabetes mellitus without cognitive impairment (T2DM) and healthy control (HC). Spearman's correlation analysis was carried out to measure the correlations between total gray matter volume with Mini‐Mental State Examination (MMSE) score (b) and Montreal Cognitive Assessment (MoCA) score (c). The data presented are mean ± SD, **p < .01; ns: no significance. One‐way analysis of variance (ANOVA) followed by a Dunn's multiple comparisons test

The total gray matter density was calculated according to the methods mentioned above. The result in Figure 3a showed that the total gray matter density was significantly decreased in the T2DM‐CI group compared with the T2DM and HC groups. However, there was no significant difference between the T2DM and HC groups. Correlation analysis results show that, in the T2DM‐CI group, the total gray matter density was significantly positively correlated with the MMSE score (*r* = 0.5059, *p* = .0016) (Figure 3b) and MoCA score (*r* = 0.4479, *p* = .0062). No correlation was found between the total gray matter density and MMSE or MoCA score in the T2DM group (data not shown).

## 4 DISCUSSION

In this study, there were no significant differences in the general clinical data (age, sex, years of education, BMI and blood pressure) among the early stage T2DM patients with and without cognitive impairment and also the HCs. The MMSE and MoCA scores were significantly decreased in the T2DM‐CI group compared with the T2DM and HC groups. The MMSE can quickly, accurately and comprehensively reflect the degree of functional impairment in intelligence and cognition in those tested. It provides a scientific and accurate basis for multiple aspects such as clinical diagnosis and treatment, as well as psychological research and is the preferred scale for dementia screening. The MMSE score of the T2DM‐CI group was 23.06 ± 2.24, which was significantly lower than 28.26 ± 0.96 of the T2DM group. Similar results were also found in the MoCA scores.

VBM is a method for automatically analyzing the density of full brain tissue based on voxel level (Ashburner & Friston, 2000). This method is objective, sensitive and repeatable and can be used to compare differences in the full brain gray matter between groups. It has been widely used in various neurological and psychiatric diseases such as mild cognitive impairment (MCI), Alzheimer's disease (AD) and Parkinson's disease to quantitatively detect abnormal microscopic changes in the brain gray matter (Karas et al., 2004; C. Xie et al., 2012; J. Zhang et al., 2015).

In recent years, many researchers have used VBM to study subtle changes in T2DM brain gray matter, with the purpose of finding the relationship between gray matter density, cognitive impairment and T2DM (Wu et al., 2017). However, the results from these studies are different, which may be related to different research sample sizes, data collection and processing methods. In this study, patients in the early stage of T2DM with and without cognitive impairment were enrolled and assessed by the MMSE and MoCA scores. All the participants in the T2DM‐CI and T2DM groups were in the early stages of the disease without requiring insulin treatment and without complications. VBM was used to measure the difference in brain gray matter density among the T2DM‐CI group, T2DM group and also the HC group to observe the characteristic brain atrophy regions in the early stages of the disease.

Compared with those in the T2DM and HC groups, the brain regions with increased gray matter density in the T2DM‐CI group were not found, which was consistent with the results of previous studies (J. Liu et al., 2017; Y. Zhang et al., 2014). The reason may be that the enrollment matched the T2DM patients in terms of age, also had brain tissue atrophy to some extent.

In detail, compared with those in the T2DM and HC groups, the density of the left triangular inferior frontal gyrus, orbital inferior frontal gyrus, operculum inferior frontal gyrus and left insular lobe was significantly reduced in the T2DM‐CI group. Insulin plays an important role in regulating glucose metabolism in the brain, and insulin receptors are selectively distributed in the cerebral cortex. Insulin is reduced during transport across the blood–brain barrier, and insulin resistance in the cortex where insulin receptors are concentrated can impair glucose metabolism in the brain and lead to atrophy of these brain regions (Srikanth et al., 2011). Y. Zhang et al. (2014) found that compared with those of the normal control group, the density of the left inferior frontal gyrus and medial frontal cortex decreased in both the T2DM group with cognitive impairment and the T2DM group without cognitive impairment. In addition, the density of the left inferior frontal gyrus and medial frontal cortex decreased significantly in the T2DM group with cognitive impairment compared with those in the MCI group, indicating that atrophy of the left inferior frontal gyrus was significantly correlated with T2DM (De Chastelaine et al., 2016; J. Liu et al., 2017; McLaren et al., 2012; Srikanth et al., 2011). This study revealed atrophy in the left inferior frontal gyrus, which was believed to be involved in the protection of memory, compared with that in the normal control group. In addition, the left inferior frontal gyrus, frontal operculum and triangular area are located in Broca's area, which is the motor language center and plays an important role in planning, language execution and complex semantic judgment. This study found that the density of the left inferior frontal gyrus in the T2DM‐CI group decreased compared with that in the T2DM group, indicating that the left inferior frontal gyrus plays a key role in cognitive impairment.

This study also found that the density of the left anterior insula in the T2DM‐CI group was lower than that in the T2DM group. As an important structure in the nervous system, the insula plays an important role in integrating social emotion, motor sensation, smell, taste and cognitive functions (Kurth et al., 2010). Furthermore, the insula is also an important node in the salience network (SN) and plays a conversion role between the central executive network and default mode network (Cai et al., 2018; R. Li et al., 2013; X. Liu et al., 2018). The anterior insula also participates in a network of structural connections with the orbitofrontal lobe, olfactory cortex, amygdala and olfactory cortex (Hoistad & Barbas, 2008). In recent years, many neuroimaging studies found that the volume of the insula, especially the left insula, decreased in MCI, AD and Parkinson's disease compared with that in normal control groups and was significantly correlated with the MMSE score (Lu et al., 2016; Zhao et al., 2015). Lu et al. (2016) found that volume atrophy of the left insula in patients with Parkinson's disease was associated with memory impairment, indicating that brain tissue atrophy in the SN could lead to memory dysfunction. In a study of memory protection in the elderly, Lin et al. (2017) found that the amplitude of low frequency fluctuations (ALFFs) in the left inferior frontal gyrus and left insula among cognitively normal elderly Apolipoprotein E (APOE) carriers was significantly increased compared with those of cognitively normal elderly non‐APOE carriers and MCI/AD APOE carriers, and importantly, decreased ALFF values in the left inferior frontal gyrus, and insula were correlated with the effect of amyloid complexes on memory impairment. Studies have shown that the frontal and insular lobes play a key role in preventing the negative effects of neurodegeneration in people at high risk of AD. In particular, the left inferior frontal gyrus and left insula lobe play key roles in protecting memory in the face of early AD pathological changes. This study also observed density atrophy of the left inferior frontal gyrus and insula lobe in T2DM patients. T2DM is an independent risk factor for AD, and in this study, the patients were in the early stages of cognitive impairment. Combined with previous VBM and functional magnetic resonance imaging (fMRI) studies, brain tissue atrophy may be caused by compensatory results due to increased cerebral blood flow and local metabolic rate (Hoogenboom et al., 2014).

It must be noted that our study has several limitations. First, analysis with the SPM software requires an artificial threshold value, which is a limitation. If the threshold value is set relatively high, the sensitivity increases, and if the threshold value is set relatively low, the specificity is not adequate. Second, the number of the samples is relatively small, and the VBM results were not adjusted. Our findings could be further verified in a larger sample size.

## 5 CONCLUSION

In conclusion, the VBM results showed that the density of left triangle part of inferior frontal gyrus, orbital part of inferior frontal gyrus and opercular part of inferior frontal gyrus and left insula was reduced in the T2DM‐CI group. Correlation analysis results showed that there was a significant positive correlation between total gray matter density and MMSE and MoCA scores in the T2DM‐CI group. Our findings suggest that total gray matter density may be an early sign of cognitive impairment in patients with T2DM.

## CONFLICT OF INTEREST

The authors declare that they have no conflict of interest.

### PEER REVIEW

The peer review history for this article is available at https://publons.com/publon/10.1002/brb3.2497

## Data Availability

Data will be made available upon reasonable request.
